# Production, Fate and Pathogenicity of Plasma Microparticles in Murine Cerebral Malaria

**DOI:** 10.1371/journal.ppat.1003839

**Published:** 2014-03-20

**Authors:** Fatima El-Assaad, Julie Wheway, Nicholas H. Hunt, Georges E. R. Grau, Valery Combes

**Affiliations:** 1 Vascular Immunology Unit, Department of Pathology, Sydney Medical School, The University of Sydney, Sydney, Australia; 2 Molecular Immunopathology Unit, Department of Pathology, Sydney Medical School, The University of Sydney, Sydney, Australia; New York University School of Medicine, United States of America

## Abstract

In patients with cerebral malaria (CM), higher levels of cell-specific microparticles (MP) correlate with the presence of neurological symptoms. MP are submicron plasma membrane-derived vesicles that express antigens of their cell of origin and phosphatidylserine (PS) on their surface, facilitating their role in coagulation, inflammation and cell adhesion. In this study, the *in vivo* production, fate and pathogenicity of cell-specific MP during *Plasmodium berghei* infection of mice were evaluated. Using annexin V, a PS ligand, and flow cytometry, analysis of platelet-free plasma from infected mice with cerebral involvement showed a peak of MP levels at the time of the neurological onset. Phenotypic analyses showed that MP from infected mice were predominantly of platelet, endothelial and erythrocytic origins. To determine the *in vivo* fate of MP, we adoptively transferred fluorescently labelled MP from mice with CM into healthy or infected recipient mice. MP were quickly cleared following intravenous injection, but microscopic examination revealed arrested MP lining the endothelium of brain vessels of infected, but not healthy, recipient mice. To determine the pathogenicity of MP, we transferred MP from activated endothelial cells into healthy recipient mice and this induced CM-like brain and lung pathology. This study supports a pathogenic role for MP in the aggravation of the neurological lesion and suggests a causal relationship between MP and the development of CM.

## Author Summary

Cerebral malaria (CM) is a potentially fatal neurological syndrome characterised by unrousable coma. Since the detection of high levels of plasma microparticles (MP) in patients with CM, it has been demonstrated that inhibition of MP production confers protection from murine CM. However, the precise mechanisms of action of these MP during CM have not been completely deciphered. In this study, we used experimental models of CM to measure the production and origins of MP over the course of infection. We found low baseline circulating MP in healthy mice and these were subsequently raised at the time of the neurological syndrome. Phenotypic analyses showed that circulating MP were predominantly from activated host cells that have previously been established to participate in CM pathogenesis. We show for the first time transferred MP impairing endothelial integrity and inducing CM-like pathology in the brain and lung of healthy animals. Our study dissects what tissues these MP localise to exert their effects, as little is known about their fate following the initial release. These data suggest a causal relationship between MP and the development of CM and also warrant further investigation into the representation of MP as a marker of CM risk.

## Introduction

Cell activation by various agonists and apoptosis trigger the vesiculation of microparticles (MP) from all cell types [1], [2], [3]. During vesiculation, phospholipids are reorganised through the translocation of inward and outward membrane lipids, whereby phosphatidylserine (PS) is exposed on the outer leaflet of the membrane [4], [5]. The budding progeny are small (0.2–1 µm) plasma membrane-derived vesicles that express antigens of their cell of origin and PS on their surface, facilitating their role in coagulation, inflammation and cell adhesion [6], [7].

Once described as inert biological bystanders MP have now emerged as novel therapeutic targets in the treatment of diseases [8], [9], [10]. Under normal physiological conditions, baseline levels of circulating MP can be detected in the blood and are thought to be involved in maintaining cellular homeostasis. However, elevated levels of MP have been implicated in several diseases [11], [12], [13], [14], [15], [16], [17], [18], including cerebral malaria (CM), in patients as well as in experimental models [19], [20], [21], [22], [23], [24], [25], [26].

CM is a multisystem multi-organ dysfunction that develops as a syndrome following *Plasmodium falciparum* infection [27]. It is characterised by the presence of sustained impaired consciousness and those surviving may develop residual neurological sequelae [28]. Despite better campaigns targeted at the eradication of malaria, the global burden persists [29], [30]. The underlying pathogenesis that drives the manifestation of CM remains incompletely understood. What is known is that the pathogenesis is multifactorial, involving the dynamic interaction between cellular sequestration, a dysregulated inflammatory response, MP production and homeostasis disruption [24], [31], [32].

Little is understood about the role of MP in CM pathogenesis, although markedly high plasma levels of circulating platelet, erythrocytic, leucocytic and in particular endothelial cell-derived MP (PMP, EryMP, LMP, EMP respectively) have been detected in patients with CM [23], [26], [33]. In murine experimental CM (eCM), the overproduction of MP is also observed, and ablation of MP vesiculation via knock-down of the ATP-binding cassette transporter A1 (ABCA1) involved in the distribution of PS, confers protection against the neurological syndrome without interfering with the infection itself [21]. Pharmacological inhibition of MP production by pantethine also confers protection from CM [8]. The above findings indicate that MP may have an active role in the development of the CM lesion and are not merely an epiphenomenon, although the precise mechanisms of action of these MP during CM have not been completely deciphered [20].

Using murine experimental models of CM [34], [35] and non-cerebral malaria (NCM) [36], [37] we characterised the production of MP over the course of *Plasmodium* infection in CM-susceptible mice, and compared their cellular origins. We adoptively transferred MP, isolated from mouse blood obtained at the time of the neurological syndrome, into the circulation of recipient mice and followed their blood clearance. Our study dissects in which tissues these MP localise to possibly exert their effects, as little is known about their fate following their initial release. Since the endothelium is an active component of the CM lesion, and EMP have been found to be elevated in human CM (hCM) [23], [38], we transferred *in vitro* generated EMP and studied their induction of pathology and clearance kinetics in healthy and infected mice. This study shows MP localised at the neurovascular lesion *in vivo* and MP transfer elicited CM-like histopathology in the brain and lung of healthy recipients, supporting a role for MP in CM pathogenesis.

## Results

### Kinetic production and characterisation of MP in CM and NCM

#### Elevated levels of total circulating plasma MP detected in CM^+^ mice

**Figure 1:**
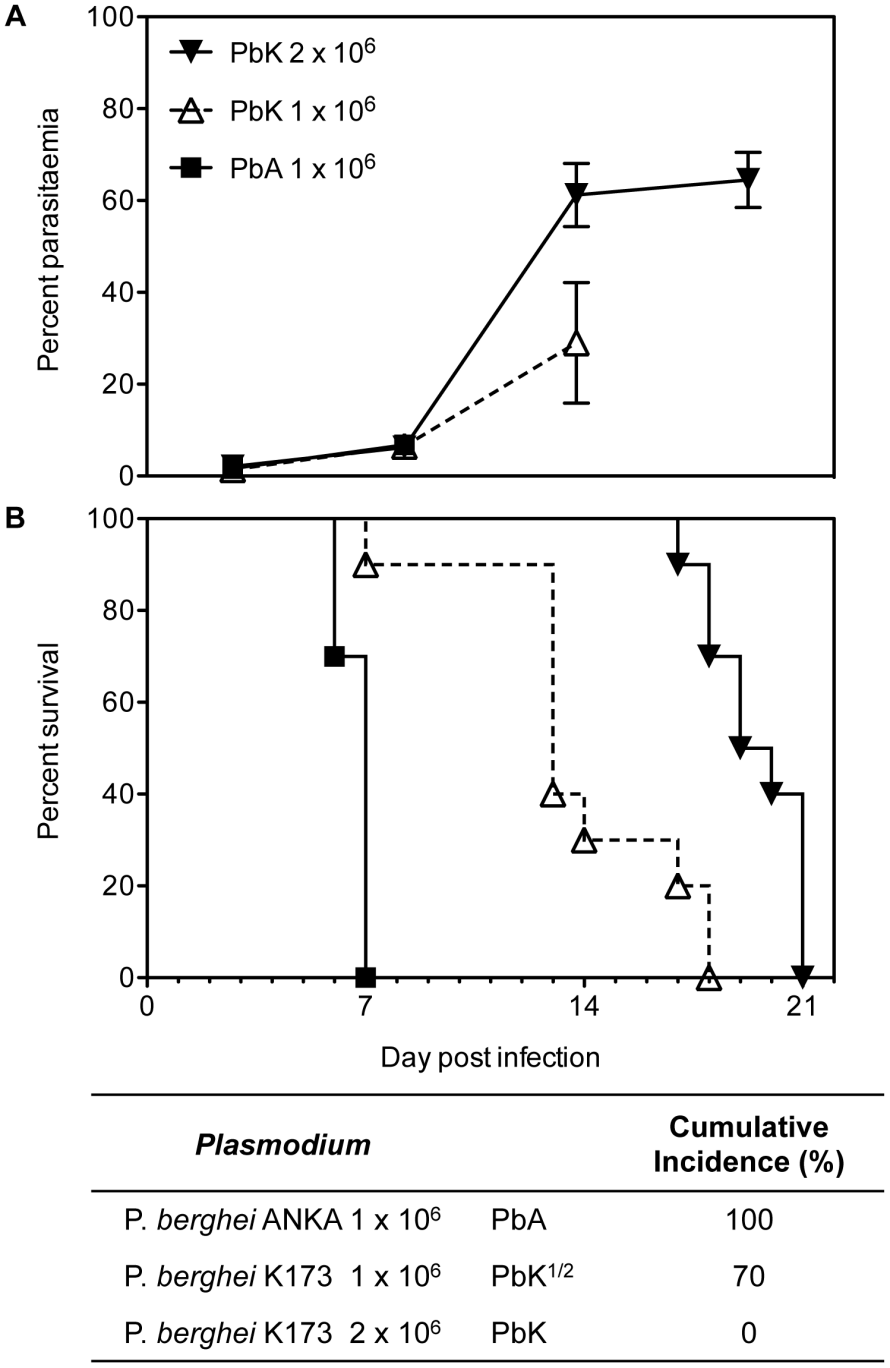
Parasitaemia and survival curves of *Plasmodium berghei*. PbK-infection at the non-encephalitogenic dose was used to model NCM (closed triangle), PbK-infection at the encephalitogenic dose (PbK^1/2^, open triangle) and PbA-infection to model CM (closed square) (n = 10). (**A**) Parasitaemia and (**B**) survival curves during the course of the infections. No difference in percentage parasitaemia on day 7 p.i by the Mann Whitney test: PbK (closed triangle) vs PbK^1/2^ (open triangle) p = 0.6286; PbK^1/2^ (open triangle) vs PbA (closed square) p = 1; PbK (closed triangle) vs PbA (closed square) p = 0.5333. Note, parasitaemia values for PbA infected mice after day 7 are not shown, since none were surviving. Parasitaemia values for PbK and PbK^1/2^ after day 7 p.i diverge. Parasitaemia is shown as mean ± SD. CBA mice (n = 10, per group) infected with PbK^1/2^ or PbA parasites develop CM, with a cumulative incidence of 70% and 100% respectively. Comparison of survival curves PbK (closed triangle) vs PbK^1/2^ (open triangle): Log-rank (Mantel-Cox) Test p<0.001 and Gehan-Breslow-Wilcoxon Test p<0.002. PbK^1/2^ (open triangle) vs PbA (closed square): Log-rank (Mantel-Cox) Test p<0.001 and Gehan-Breslow-Wilcoxon Test p<0.001. PbK (closed triangle) vs PbA^1/2^ (closed square): Log-rank (Mantel-Cox) Test p<0.001 and Gehan-Breslow-Wilcoxon Test p<0.001.

In our study, PbK infection at the non-encephalitogenic dose (PbK) was used to model NCM, PbK infection at the encephalitogenic dose (PbK^1/2^) and PbA infection (PbA) to model CM (Figure 1). In our models, 100% of mice inoculated with PbA and 70% of mice inoculated at the encephalitogenic dose with 1×10^6^ PbK developed CM within the neurological phase. Mice infected at the non-encephalitogenic dose (2×10^6^ PRBC) of PbK developed NCM and no cerebral complications. No significant differences in the evolution of parasitaemia were observed between the 3 infections on day 7 p.i (PbK, PbK^1/2^ and PbA, Fig. 1A). Parasitaemia values for PbK and PbK^1/2^ after day 7 p.i diverge.

**Figure 2:**
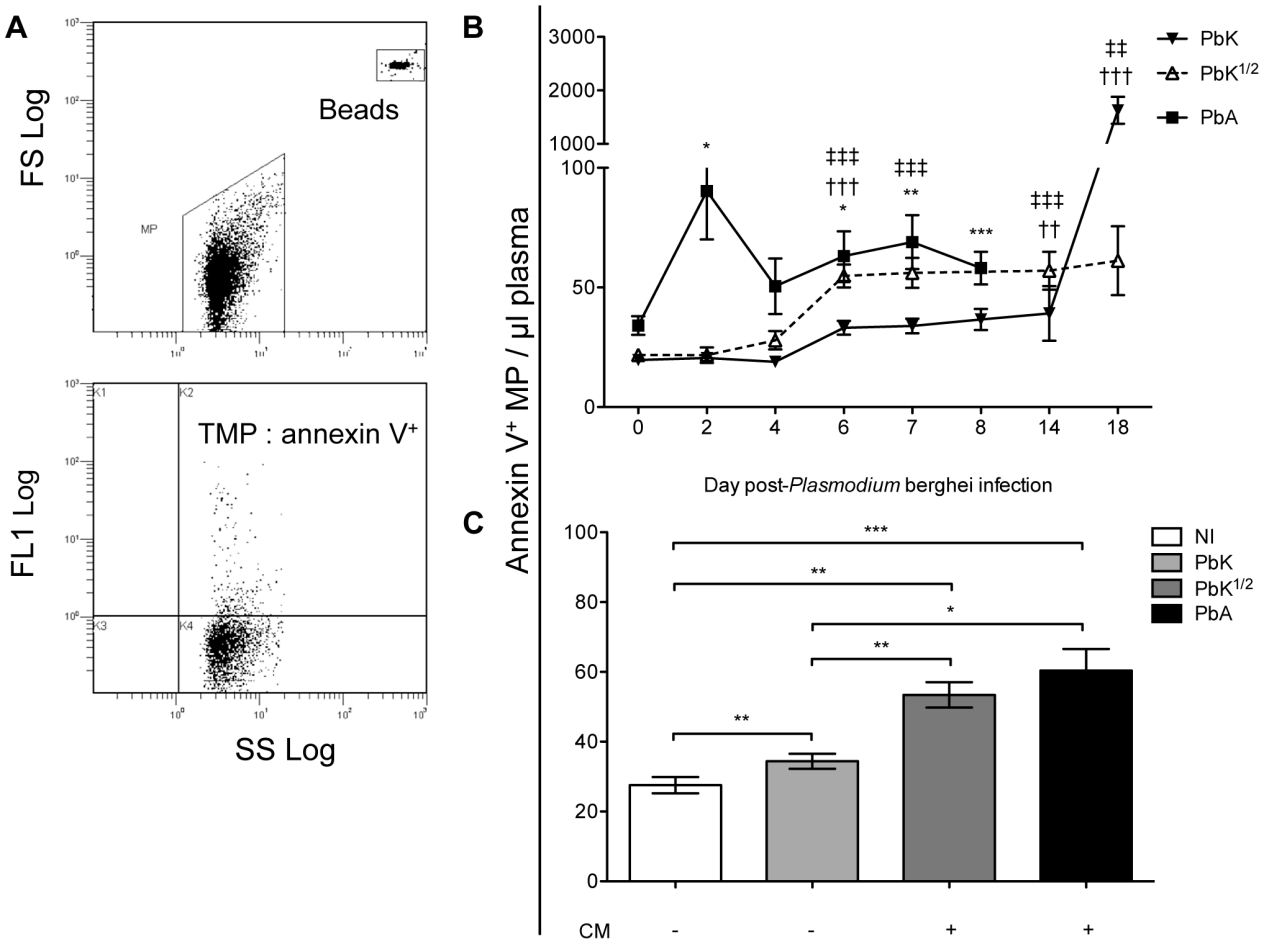
Total plasma microparticle levels during *Plasmodium berghei* infection. (**A**) Cytogram representation of total Annexin V^+^ MP per µL of plasma in infected CBA mice. MP were gated for their size and further analysed for positive Annexin V^+^ populations. Calibrated fluorescent latex beads of a known size and concentration were used as an internal standard to calculate MP levels. (**B**) Increase in total Annexin V^+^ MP following *Plasmodium berghei* infection. Mice infected with 1×10^6^ PbA (closed square) display a biphasic production of MP, peaking at day 2 and day 7, which is not observed in mice infected with PbK (closed triangle) or PbK^1/2^ (open triangle). MP levels are plotted in MP/µL of plasma; 1-way ANOVA, Kruskall-Wallis & Dunns post-test *p<0.05, **p<0.001, ***p<0.0001. Annexin V^+^ labelling was performed on 5 PFP samples from each mouse. Data shown from n = 10 PbK (closed triangle) and n = 5 PbK^1/2^ infected (open triangle) mice on day 0, 2, 4, 6, 7, 14 and 18. Data shown from PbA infected mice (closed square) n = 11 day 0, n = 10 day 2, n = 10 day 4, n = 12 day 6, n = 6 day 7, n = 5 day 8. (**C**) Annexin V^+^ MP per µL of plasma at time of CM onset. CM^+^ mice (i.e. Infected with PbK^1/2^ (dark grey) or PbA (black)) have higher levels of circulating MP than CM^−^ mice (i.e. non-infected (white) and PbK infected mice (light grey)). Data from PbK^1/2^ infected mice that did not display signs of CM are not shown. Mann Whitney t-test *p<0.05, **p<0.001, ***p<0.0001.

We collected PFP following *P. berghei* injection and quantified circulating Annexin V^+^ MP over the course of infection. MP were gated for their size and further analysed for Annexin V positive populations using flow cytometry (Fig. 2A). In healthy mice, low baseline levels of MP were detected (mean ± SEM MP/µL, 27.6±2.3) and *P. berghei* infection triggered a rise in plasma Annexin V^+^ MP levels (Fig. 2B). In PbA infected mice, numbers of Annexin V^+^ MP peaked on day 2 (90.3±20.3) and during the neurological phase (60.3±6.2). High levels of Annexin V^+^ MP were detected in the plasma of PbK^1/2^ infected mice on days 6 and 7 p.i, although this profile was different to PbA infection, notably the absence of the peak at day 2 and the presence of the peak at day 18 for the mice which did not develop CM. Mice infected with PbK had a rise in MP levels on days 6 p.i. and on day 14 and 18 p.i (33.2±2.9; 39.2±11.5; 1627.8±251.7).

Plasma MP numbers were compared at the time of CM onset (day 6–8) between the groups of infected mice, i.e. PbK-, PbK^1/2^- and PbA (Fig. 2C). Annexin V^+^ MP were significantly increased in mice showing signs of CM (PbA infected 60.3±6.2; PbK^1/2^ infected 53.4±3.6), when compared to healthy controls (27.6±2.3) and infected mice not showing cerebral involvement (PbK infected mean, 34.4±2.4; PbK^1/2^ infected mice with NCM are not shown in figure). Interestingly, infected mice that develop CM displayed higher levels of plasma MP than those that did not.

#### Elevated endothelial, platelet and erythrocytic MP at time of CM onset

**Figure 3:**
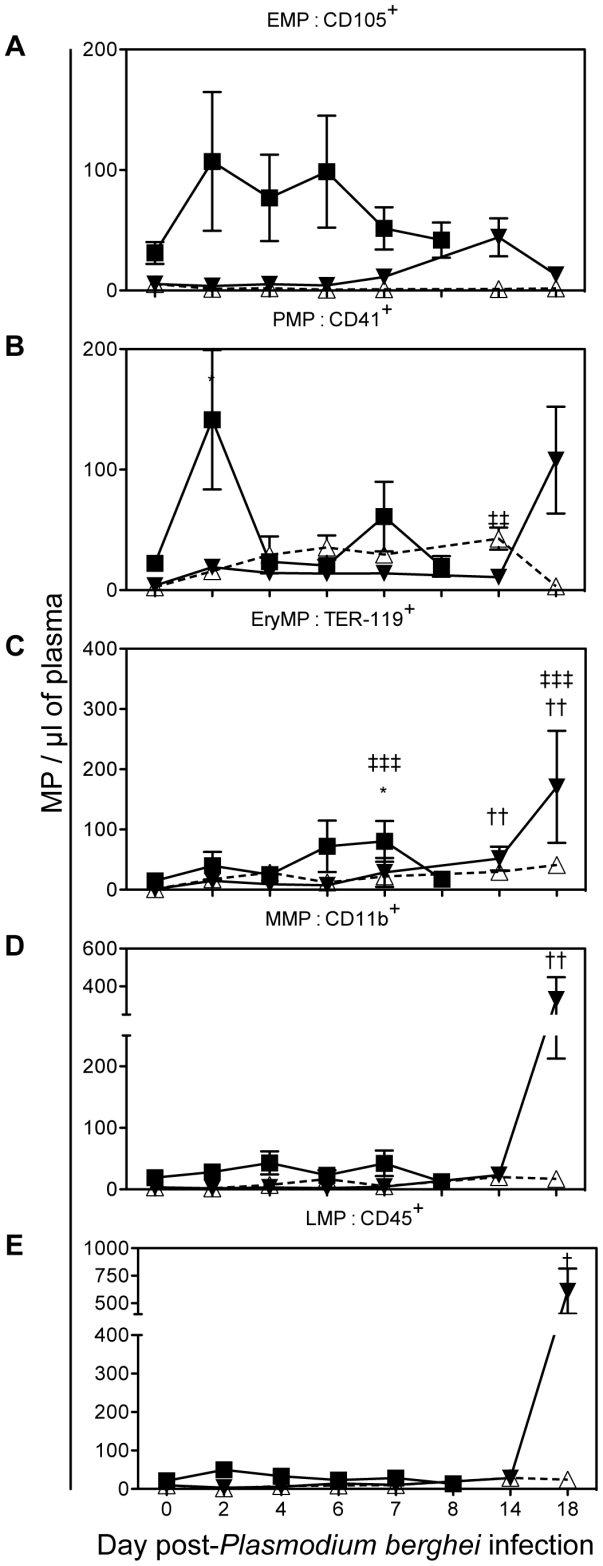
Cell-specific MP in plasma during the course of *Plasmodium berghei* infection of CBA mice. PbK (closed triangle), PbK^1/2^ (open triangle) or PbA (closed square) (**A**) PMP/µL plasma; (**B**) EMP/µL plasma; (**C**) RMP/µL plasma; (**D**) MMP/µL plasma; (**E**) LMP/µL plasma. Data shown from *n = 10* PbK- (closed triangle) and *n = *5 PbK^1/2^ infected (open triangle) mice on day 0, 2, 4, 6, 7, 14 and 18. Data shown from PbA infected mice (closed square) *n = *11 day 0, *n = *10 day 2, *n = *10 day 4, *n = *12 day 6, *n = 6* day 7, *n = 5* day 8 and represented as mean ± SEM *p<0.05, **p<0.001, ***p<0.0001. Inconsistencies in sample group size are due to the fragile health status of mice during the period of morbidity.

To evaluate whether *Plasmodium* infection could directly or indirectly modify the cellular origin of MP present in the plasma, we studied cell-specific MP level over the course of infection by performing double staining for each cell marker (Fig. 3) and PS. Although not significant, there was a trend for CD105^+^ EMP to be elevated in the PbA infected group between day 2 to 6, at day 14p.i. in the PbK infected group and remained unchanged in the PbK^1/2^ infected group. High levels of CD41^+^ PMP were seen on day 2 p.i in the PbA infected group (141.4±57.9) and on day 14 in the PbK^1/2^ infected group (NCM) (42.8±9.2). On day 7 p.i, TER119^+^ EryMP were elevated in PbA infected mice (80.3±33.8) compared to healthy (7.1±2.4) and PbK infected mice (22.3±3). During the late stage of infection, day 18, PbK infected mice exhibited higher levels of TER119^+^ EryMP (170.9±93.0), CD11b^+^ MMP (330.4±117.7) and CD45^+^ LMP (609.3±205.1).

**Figure 4:**
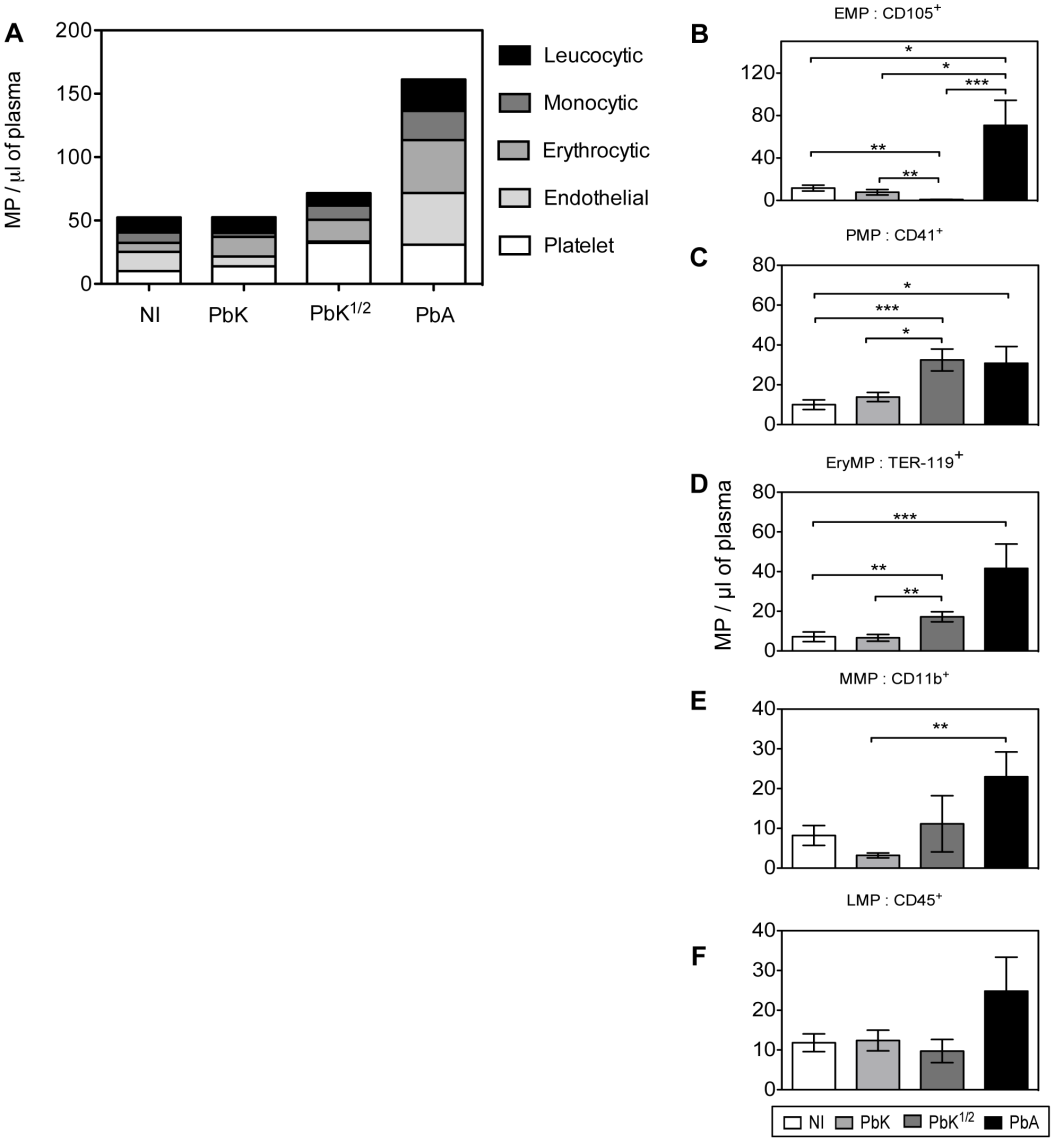
Cell-specific MP in plasma at CM onset in CBA mice. (**A**) Levels of total cell-specific MP in plasma at time of CM onset: platelet-MP (PMP, white), endothelial-MP (EMP, light grey), erythrocytic-MP (EryMP, dark grey), monocytic-MP (MMP, black), leucocytic-MP (LMP, striped). (**B–F**) Cell-specific MP levels per µL of plasma at time of CM onset: non-infected (white bar), PbK (light grey bar), PbK^1/2^ (dark grey bar), PbA (black bar). **B.** PMP. **C.** EMP. **D.** RMP. **E.** MMP. **F.** LMP. Data shown from *n = *25 healthy, *n = *20 PbK infected and *n = *10 PbK^1/2^ infected mice showing signs of CM on day 6 and 7 p.i. and from *n = *23 PbA infected mice collected on day 6, 7 and 8 p.i., and represented as mean ± SEM. *p<0.05, **p<0.001, ***p<0.0001.

We studied the levels of cell-specific MP in the plasma at the time of CM onset (Fig. 4A; PbK^1/2^ infected mice with NCM are not shown in the figure). We found that EryMP (41.6±12.3), EMP (41±13.3) and PMP (30.9±8.3) were most numerous in PbA infected mice at the time of CM onset (Fig. 4A). In the PbA groups, the levels of EMP, PMP and EryMP increased from 15.3±4.4, 10.0±2.4 and 7.1±2.4, respectively in the controls, to 41±13.3, 30.9±8.3 and 41.6±12.3 at the onset of CM, respectively (Fig. 4B–D). In PbK^1/2^ infected mice, the levels of PMP and EryMP increased at time of onset (32.5±5.5; 17.2±2.5) with a decrease observed in EMP (0.9±0.2). No statistical difference was observed with cell-specific MP between healthy controls and PbK infected mice.

### *In vivo* transfer and detection of MP

#### Rapid clearance of transferred MP in the circulation following adoptive transfer

**Figure 5:**
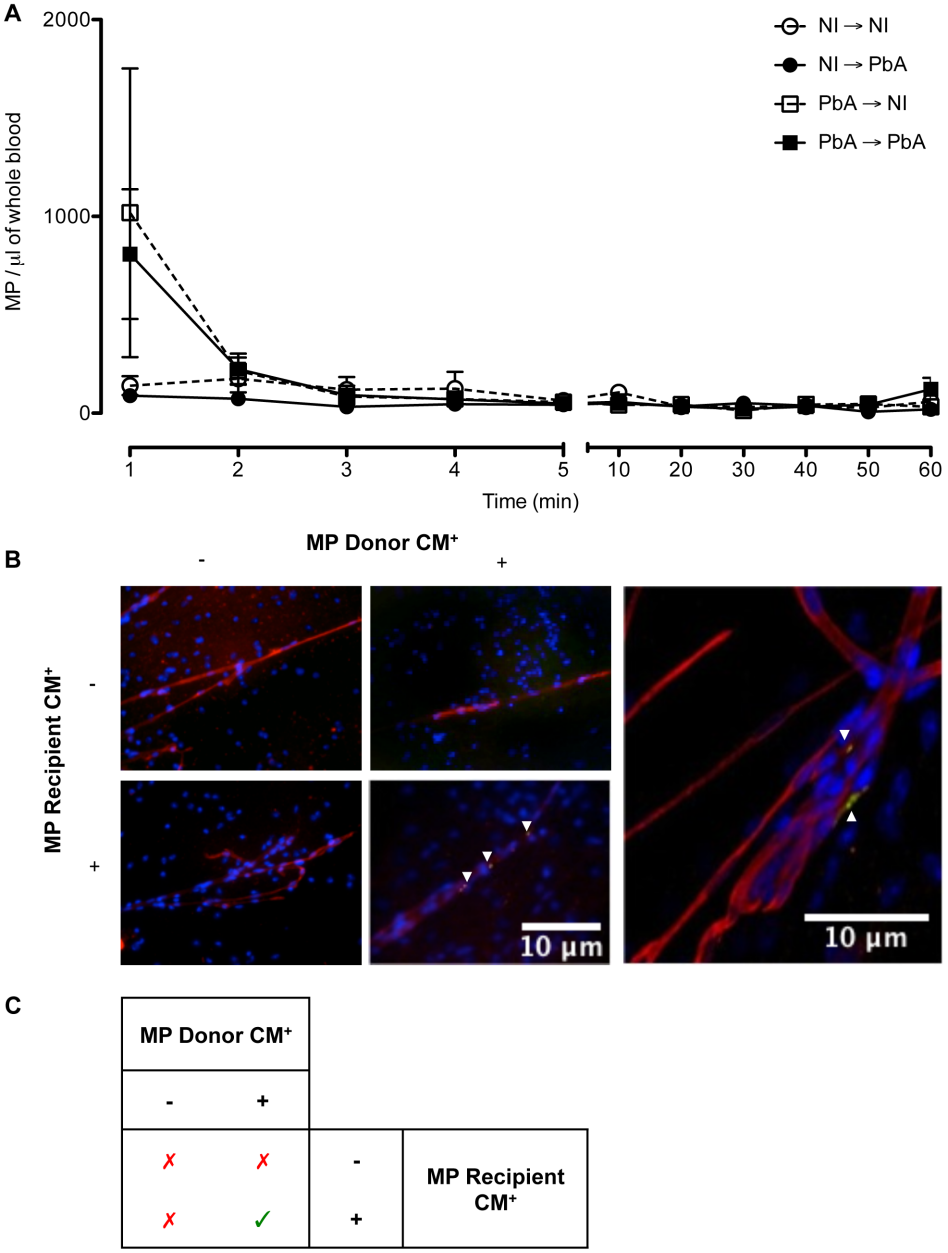
Detection, clearance, and tissue distribution of MP following adoptive transfer. (**A**) Clearance of transferred MP within circulation following adoptive transfer. The presence of transferred PKH67-labeled MP was detected via flow cytometry in the blood of recipient mice. MP were detectable immediately following intravenous injection; healthy recipients (circles) cleared the MP quicker than PbA infected recipients (squares). Data are mean ± SEM from *n* = 3 per group. (**B**) Microparticles localised in brain microvessels of CM^+^ recipient mice. Smears were prepared from healthy and CM^+^ mice, recipients of PKH67-labelled MP (green) purified from healthy or CM^+^ donor mice (*n = *3). MP were activated *in vitro* with calcium ionophore (A23187), a known vesiculating agonist, labelled, intravenously transferred and allowed to circulate for 1 h. Brain smears were fixed and counter-stained with Texas Red Lectin and DAPI to identify vessels (red) and nuclei (blue), respectively. MP from CM^+^ donors can only be detected in the microvessels of CM^+^ recipient mice (arrows). Imaged on Olympus IX71. Insert: MP from CM^+^ donor lodged in CM^+^ brain vessel, imaged using oil immersion ×60 on Olympus FluoView FV 1000 confocal microscope. Arrows indicate MP lining the endothelium amongst other cells and trapped in birfurcation of vessels. (**C**) Summary of MP localisation in cerebral vessels. MP from CM^+^ donors can only be detected in the microvessels of CM^+^ recipient mice, as indicated by green tick.

Fluorescently labeled MP, purified from the plasma of healthy and PbA infected donor mice, were adoptively transferred into healthy or PbA infected recipient mice. The presence of these MP was assessed in the blood of recipient mice via flow cytometry over 60 minutes post injection. MP from healthy donors were detectable in significantly lower numbers than those from PbA infected donors. Injecting MP from PbA infected donors induced an acute rise of MP in recipient mice (mean ± SEM MP/µL, 1018.5±733.49 healthy recipient, 808.23±329.75 PbA recipient) that cleared within 2 minutes following injection (Fig. 5A).

#### MP from PbA infected donors localised in cerebral microvessels of PbA infected recipients

Brain smears were prepared from all recipient mice. Fluorescence microscopic analysis showed that, after transfer, only MP from PbA infected mice showing signs of CM (CM^+^) were found within the vessels of the brain of CM^+^ recipient mice, but this was not evident in NCM mice. MP were found to be lodged along the endothelium within the lumen and at the bifurcation of some but not all microvessels. When the recipient mice were healthy, no MP could be visualised within the cerebral microvessels (Fig. 5B, Table 1). Similarly, transferred MP from healthy donors were absent in the vessels of both healthy and CM^+^ recipient mice (Fig. 5B, Table 1).

**Table 1:** Qualitative summary of PKH67-labelled MP distribution within the tissue of recipient mice following adoptive transfer.

	Donor	Recipient	MP present
**Spleen**	healthy	healthy	+
	healthy	PbA	+
	PbA	healthy	++
	PbA	PbA	+++
**Kidney**	healthy	healthy	−
	healthy	PbA	−
	PbA	healthy	−
	PbA	PbA	+
**Brain**	healthy	healthy	−
	healthy	PbA	−
	PbA	healthy	−
	PbA	PbA	+
**Lung**	healthy	healthy	−
	healthy	PbA	−
	PbA	healthy	−
	PbA	PbA	+/−
**Liver**	healthy	healthy	−
	healthy	PbA	−
	PbA	healthy	−
	PbA	PbA	+/−
**Heart**	healthy	healthy	−
	healthy	PbA	−
	PbA	healthy	−
	PbA	PbA	−

#### Transferred MP are trapped in both PbA infected and healthy recipient spleen

To determine the major route of clearance for the adoptively transferred MP we imaged the spleen, kidney, liver, lung and heart of recipient mice. Observations made on all combinations MP-donor/MP-recipient and the presence of MP within these organs are represented in Table 1 (and in Fig. S1).

MP from both healthy and PbA infected donors were present in the red pulp of the spleen of all recipient mice (Table 1). CM^+^ MP were distributed in CM^+^ kidney and to a lesser extent in CM^+^ lung and CM^+^ liver. No MP were present in the heart, an organ without any known CM pathology (Table 1).

#### Transferred EMP induced several histopathological anomalies in the brain and lungs of recipient mice

**Figure 6:**
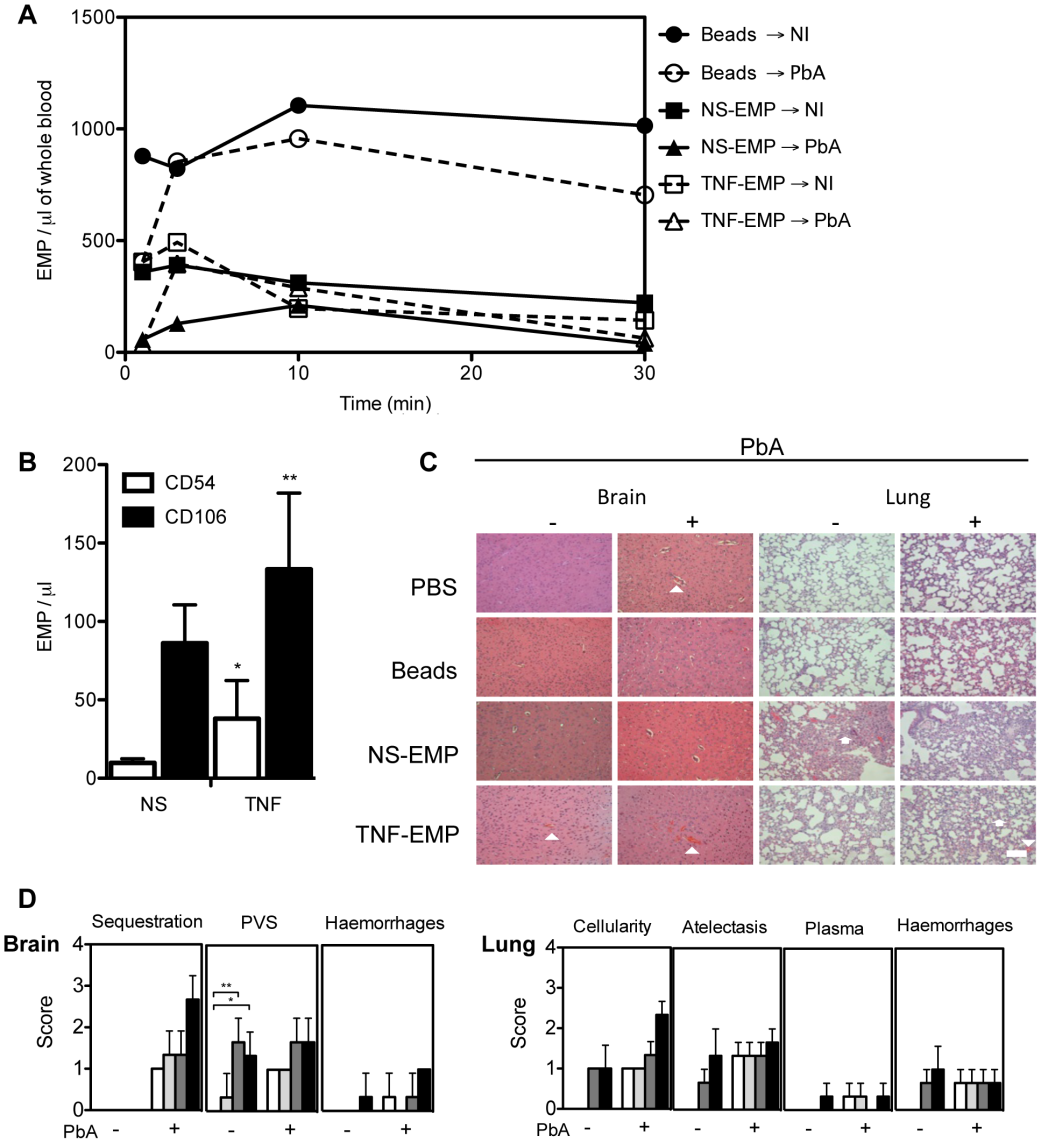
Transferred EMP generated *in vitro* induce CM-like pathology in healthy mice. (**A**) Detection of PKH-67 labelled EMP in the blood of recipient mice following transfer. Purified EMP (400×10^3^/mouse) from TNF stimulated and resting mouse brain microvascular endothelial cell line B3 harvested *in vitro*, were transferred into PbA infected and healthy recipient mice. Clearance of fluorescently labeled EMP from the blood was monitored by flow cytometry. Latex beads (400×10^3^/mouse, control) can be detected in constant circulation up to 30 minutes post transfer. Data represent the mean from *n* = 3. Closed circle/solid line indicates beads transferred into healthy mice, open circle/interrupted line indicates beads transferred into infected mice, closed square/solid line indicates NS- EMP transferred into healthy mice, closed triangle/solid line indicates NS-EMP transferred into infected mice, open square/interrupted line indicates TNF-EMP transferred into healthy mice and open triangle/interrupted line indicates TNF-EMP transferred into infected mice. (**B**) Detection of CD54 and CD106 on *in vitro* generated MP derived from mouse brain microvascular endothelial cells. Graph shows the expression of CD54 (white bar) and CD106 (black bar) on resting and TNF-stimulated EMP. Data are mean ± SD, *p<0.05. The numbers of CD54^+^ and CD106^+^ EMP in the supernatant increased following TNF-stimulation (mean ± SEM MP/µL; CD54: 9.850±1.064 to 38.050±9.925; 74.11% CD54^+^ shift CD106: 86.183±9.981 to 133.250±19.860; 35.32% CD106^+^ shift). (**C**) Representative haematoxylin-eosin brain and lung sections from healthy and PbA infected mice treated with PBS or EMP (*n = *3) on day 6 p.i. Morphometric analysis reveals that EMPs induce significant pathology in healthy brain and lung. In the brain, the arrows heads indicate areas of engorged vessels and haemorrhage. In the lung, multifocal lesions, abnormal cellularity in the alveolar septa, plasma in alveoli and haemorrhage. Treatment with EMP in healthy mice induces haemorrhage in the brain (white arrow head) and increased cellularity (white arrow head) and atelectasis (white arrow) in the lung of recipient mice. Such pathology is not observed in the mice treated with PBS or beads alone. Scale bar (bottom left) indicates 40 µm. (**D**) Effects of *in vitro* generated EMP on healthy and PbA infected brain and lung in the following treated groups: PBS (white bar), beads (light grey), NS-EMP (dark grey) and TNF-EMP (black). Representative Haematoxylin-Eosin images (*n = *3) per group were scored for histopathological signs (Table 3).

EMP purified from resting or TNF-stimulated MVECs *in vitro* were labelled and transferred into healthy or PbA infected mice to investigate their role in the CM lesion. EMP could be detected circulating in the first few minutes post-transfer compared to the control microsphere beads that continued to circulate longer (Fig. 6A) and could be detected for up to 60 minutes (Fig. 6A, data shown only until 30 min).

To identify possible markers that could be facilitating the interaction of EMP at the site of the lesion, the surface phenotype of EMP was determined. We found that CD54 and CD106 (Fig. 6B) were present on EMP from resting cells and the numbers of CD54^+^ and CD106^+^ EMP in the supernatant increased following TNF-stimulation (mean ± SEM MP/µL; CD54: 9.850±1.064 to 38.050±9.925; CD106: 86.183±9.981 to 133.250±19.860). To characterise the extent of pathology induced by the transfer of EMP, we examined multiple haematoxylin-eosin stained representative brain and lung sections from each group sampled on day 7 post-infection (Fig. 6C–D) and scored the 3 main pathological features of CM, i.e., sequestration, enlarged perivascular spaces (PVS) and haemorrhages. Enlarged PVS were present in brain sections of healthy mice injected with NS-EMP or TNF-EMP and not in sections sampled from mice treated with PBS or beads (p < 0.05). Evidence for vessel disruption and haemorrhages were seen in brain sections from uninfected mice injected with TNF-EMP. These histological changes closely resembled brain sections of PbA infected mice treated with PBS and control beads. In the EMP-injected uninfected lung, we found signs of pulmonary inflammation including hyperplasia of alveolar epithelium and the presence of increased numbers of leucocytes in alveolar septa (Fig. 6C). Both NS-EMP and TNF-EMP induced thickening of the alveolar septa, atelectasis and haemorrhages, closely resembling the ARDS-like pathology usually seen in PbA infected mice. Injection of beads or PBS did not appear to alter lung histology.

## Discussion

This study investigates the levels and cellular origin of MP produced during murine malaria and for the first time describes the clearance and fate of CM^+^ MP *in vivo* as well as the pathogenicity of EMP. Using CM-susceptible mice, we showed that *P. berghei* infection induces a rise in total circulating MP, although distinct differences exist in the cellular origin and production profiles of MP in mice developing CM versus NCM. We found that MP from CM^+^, but not healthy donors, adoptively transferred *in vivo* can be detected at the site of the lesion, sequestered amongst other cells within the cerebral vessels of CM^+^ recipient mice. We also showed that transferred EMP,induce CM-like pathology in the brain and lung of recipient mice, supporting a role for MP in the exacerbation of CM.

Ablating the increase of MP numbers either genetically [21] or pharmacologically [8] confers protection against murine CM. Elevated levels of plasma EryMP [33] and EMP [23], [38] in malaria patients correlate with severity and are particularly restricted to those with cerebral involvement. This also has been shown in studies on intracerebral haemorrhage, whereby higher MP levels correlate with coma and poor clinical outcome in patients [39] [40]. Although suggestive of a role in the pathogenesis of CM, the existing data on MP in CM does not completely support a role for MP in the worsening of CM nor do they substantiate it solely as a predictive marker for CM.

Elevated levels of circulating Annexin V^+^ MP were detected in the plasma of CM^+^ mice (i.e. infected with PbA or PbK^1/2^) at the time of neurological development. This finding confirms what has already been observed upon CM onset in experimental and hCM [21], [38]. Our study extends from this and follows the production of MP over the course of infection, not just at CM onset. Interestingly, a biphasic production of MP was detected in the plasma of PbA infected mice, peaking during the early stages of infection and also at CM onset. These waves of MP coincide with the perpetuated cycle of endothelial activation, the production of cytokines and chemokines, the upregulation of adhesion molecules and the binding of vascular cells to microvessels [41]. Although the first wave of MP was absent in PbK^1/2^ infection, mice with high MP levels during the neurological phase did develop CM. MP overproduction was absent in mice that did not develop cerebral signs during the time of CM onset in PbA mice (i.e. PbK infected or 30% of the PbK^1/2^ infected). This suggests that MP may play a role in the development of the neurological syndrome.

At the time of CM onset, cell-specific MP numbers were higher in PbA infected mice than in PbK- and PbK^1/2^ infected animals or healthy controls. The sum of positive MP for single staining of cell markers gave a closer approximation of total circulating MP, as Annexin V staining indicates only PS-positive MP. MP can be PS negative or have low undetectable PS on the surface and remain unbound to Annexin V [20], [42]. We found that plasma PMP, EMP, EryMP and MMP were most elevated during the neurological phase in PbA infected mice. Previous studies have shown a dramatic increase in EMP, EryMP and PMP in patients presenting with CM [23], [33], [38]. In CM, the neurovascular lesion is comprised of sequestered vascular cells such as platelets, erythrocytes and leucocytes within the endothelial lining of microvessels [32], [43], [44]. It is not surprising that the sequestered cells also produce the most predominant MP populations detected. The development of CM is attributed to the cascade of events preceding the sequestration of cells and, consequently, the mechanical occlusion [31], [43], [44], [45]. The 70% of PbK^1/2^ infected mice that developed CM displayed comparable levels of total Annexin V^+^ MP levels to PbA infected mice, although their origins were predominately from platelets and erythrocytes. No significant differences were detected between the levels or proportions of cell-specific MP detected in PbK infected and healthy mice between days 6 and 14.

Our data show that during the acute stage of PbA-infection, a peak of PMP can be detected, and this is absent in the PbK- and PbK^1/2^-infections. This finding is consistent with the substantial loss of platelets as MP in the acute phase of the PbA-infection [46] and may correlate with the depth and duration of coma [38]. Thrombocytopenia is associated with poor prognosis in both human and experimental CM [46], [47] and higher platelet accumulation has been observed in cerebral microvessels in both human [48] and eCM [49], [50]. *In vitro*, platelets enhance the binding of infected erythrocytes (IE) to the cerebral endothelium [51] and their MP progeny are able to adhere preferentially to IE and also to the cerebral endothelium [19], [52]. PMP pathogenic potential is attributed to their mobility and their access via blood flow to other vascular cells [53]. Although this finding supports the platelet adhesion hypothesis, the absence of this first wave of PMP in PbK^1/2^ infection is interesting. Nothing is known about thrombocytopenia or PMP in PbK^1/2^ infection. The majority of mice develop CM^+^ despite the absence of the first wave of PMP; nevertheless, at CM onset, higher titres of PMP are detected.

EryMP were elevated in CM^+^ mice, both in PbA- and PbK^1/2^ infected mice, at CM onset. This finding is supported by human studies, whereby EryMP numbers are increased in patients with *P. falciparum* infections, even after antimalarial drug treatment [33] and also in patients with severe malarial anaemia [38]. In contrast, EryMP numbers are lower in patients with *P. vivax* and *P. malariae* malaria, similar to what is observed with the PbK infected mice in our study [33]. Interestingly, the PbK infected group, with no evidence of cerebral complications, had an overproduction of MP from erythrocytes and leucocytes during the later stage of infection. The evolving hyperparasitaemia in these mice leads to the destruction of IE and the gradual rupture and fragmentation of fragile erythrocytes increases the level of EryMP and cellular debris [33]. Higher levels of LMP could account in that case for the activation of the cells involved in the destruction of erythrocytes and their removal from the circulation.

Adoptively transferred CM^+^ MP are detectable in recipient mice but quickly subsided, indicating clearance from circulation. Some of these MP were found to be arrested in cerebral microvessels of CM^+^ recipients and also in the spleen, kidney and, to a lesser extent, the lung and liver. Recent studies in humans have shown that parasites induce the loss of endothelial protein C receptors in the cerebral microvessels, leaving them vulnerable to enhanced local thrombin generation and coagulopathy [54]. We know from previous murine studies that MP are procoagulant and proinflammatory [21], [43], [55], thus it is not surprising that MP are found lodged in the cerebral microvessels of infected recipients. No MP were detected in the heart of recipients. MP from both healthy and CM^+^ donors were also detected within the spleen of recipient hosts, suggesting that this organ could be a site of MP trapping and clearance from the circulation independent of the infection. It is possible that the spleen filters the PS^+^ MP in a similar way to PS^+^ cells in malaria [56] and Kupffer cells in the liver could remove EryMP as shown by Willenkens et al., [57], although further studies are required to elucidate this in our system.

Little is known on the mechanisms underlying the clearance of plasma MP from circulation *in vivo*. In our study, the disappearance of PKH67-labelled CM^+^ MP occurred within 5 minutes. In a rabbit model, no PMP were detected in the circulation at 10, 30 or 60 minutes post injection [58]. In contrast, transferred PMP, isolated from platelet concentrates from the peripheral blood of single donor patients, circulated for markedly longer with a half-life of 5.8 hours (Annexin V^+^) and 5.3 hours (CD61^+^) [59]. The authors attribute the elevated levels and the longer half-life of circulating MP to the infused platelets producing more MP in circulation [59]. Another possible explanation is that MP may clear the circulation and reach their target sites quicker in rabbit models, and also in mice, due to their faster heart rate [58]. The authors also suggest that the half-life is an overestimation, and the observations in the rabbits miss the initial rapid clearance of MP [58]. Furthermore, *in vitro* assays support the idea that MP can be detected in blood when there is no mechanism to remove them [58]. In pathological states, the continuous production of MP overrides the mechanisms of rapid clearance, hence elevated levels can be detected.

All cells are able to produce and release MP, although the emerging progeny of MP are heterogeneous and do not share the same properties. EMP represent the most abundant MP detected in pathologies that arise due to vascular injury or endothelial dysfunction although not all their roles are noxious [60], [61]. EMP numbers are elevated in patients with conditions in which the endothelium is injured and/or the endothelial barrier is compromised, including sickle cell disease, Alzheimer's disease, metabolic syndrome, hypertension, atherosclerosis and chronic obstructive pulmonary disease [62], [63], [64], [65], [66], [67]. EMP were first described *in vitro* [6] and high numbers were detected in CM patients presenting with coma [23]. Lower numbers of EMP and concentrations of TNF were detected in mice protected against CM during PbA infection [21]. Studies *in vitro* and in the murine model of CM indicated that EMP have similar procoagulant and pro-inflammatory potentials to, and express the same repertoire of antigens as, their corresponding mother EC [6], [21]. In CM, the endothelium is both a target and an effector in disease pathogenesis [24]. The direct role of EMP in inducing brain and lung pathology in murine models of CM has not been described. We addressed the hypothesis that EMP may be pathogenic in the CM lesion by transferring TNF-generated EMP into healthy and infected mice. Our findings demonstrate that transferred TNF-EMP can induce histopathological signs that are compatible with endothelial leakage leading to cerebral and pulmonary oedema and haemorrhage in healthy mice [68]. Control inert microsphere beads did not induce any change and remained in circulation for 60 minutes, compared to EMP that were cleared within the first few minutes post transfer, supporting the idea that the clearance of the MP is a physiological phenomenon that is mediated by receptors present at the surface of MP.

Exactly how EMP alter endothelial integrity in CM is unclear. Flow cytometry revealed that our EMP express endoglin, CD54 and CD106. Soluble endoglin (sCD105) overexpression is linked to typical systemic and vascular inflammation states such as pre-eclampsia and HELLP syndrome [69]. It is possible that endoglin-bound MP may have a role in inducing vascular injury. Endoglin is an RGD membrane protein acting as transforming growth factor-β accessory receptor and has been implicated in leucocyte recruitment and extravasation [70] and more recently in septic shock-induced disseminated intravascular coagulopathy [71]. In CM, CD54 and CD106 are established markers of EC injury and enable tethering and sequestration of cells to the endothelium [31], [43], [72]. TNF increased the expression of CD54 and CD106 on the EMP, consistent with other studies [25], suggesting a possible mechanism by which MP interact with the endothelium to induce injury.

Transferred EMP induced atelectasis in lungs from healthy recipient mice and increased alveolar cellularity, resembling the pathology seen in CM^+^ lung. EMP were shown to induce endothelial dysfunction, promote vasodilation, pulmonary oedema and acute lung injury in pathophysiological concentrations [73]. EMP sequester in lung tissue and elicit an immune response *in vivo* by increasing cytokine production leading to neutrophil recruitment [73], [74]. It is plausible that EMP initiate a cascade of events, beginning with the production of cytokines, that prime the endothelium thereby ultimately impairing vessel functions and resulting in tissue damage.

Taken together, our findings offer new evidence for a causal relationship between MP and the pathogenesis of CM. To our knowledge, this is the first time that MP have been localised at the neurovascular lesion *in vivo* and that their transfer elicited histopathology in the brain and lung of healthy recipients. We confirm that elevated levels of total MP are present at CM onset, predominantly from activated host cells that are known to participate in CM pathogenesis. Specifically, in CM, the early peak of total MP and PMP differentiated the kinetic production profile from NCM and could potentially be an indicator of prognosis. We showed that MP are rapidly cleared from circulation, and that some remain sequestered in organs, such as the brain and spleen. The EMP in this study carry VCAM-1 and ICAM-1 on their surface, which are upregulated following cytokine stimulation, potentially mediating their role in the worsening of CM.

During CM, activation of cells by parasite moieties, toxins, cytokines and/or cell death, delivers MP into the circulation. We propose that the interactions between MP, endothelium, circulating host vascular cells and their released circulating soluble factors influence the course of infection leading to the development of CM. Since the first human studies on MP in CM [23], [38], there has been growing interest in exploring the potential of MP as biomarkers for both diagnosis and follow-up and therapeutic targets since they represent both a consequence of, and contributor to, CM. The plasma membrane acts as the primary sensor to its external environment, thus, discriminating the cellular origin of MP may indicate what tissues or cells are undergoing activation or damage. Besides detection of malarial retinopathy, which is yet to be included as standard assessment for severe malaria, a clinical challenge still exists to distinguish CM from other encephalopathies [75]. Diagnosis relies on *P. falciparum* parasitaemia and impaired consciousness with the exclusion of other potential causes of severe disease, which in malaria-endemic areas is difficult to achieve due to the prevalence of asymptomatic parasitaemia and the lack of high-level diagnostic testing. Misdiagnosis is common and there is a need for reliable CM specific diagnostic and prognostic biomarkers [76]. This would support the promise of MP as clinical probes for CM and help provide targeted care of malaria patients at imminent risk of organ damage or cerebral complications as indicated by their detected MP [11].

In addition, molecules such as Pantethine that inhibit MP release have conferred protection *in vivo* and may be suitable candidates for an adjunct neuroprotective treatment of CM [8]. If the delay of CM onset *in vivo* is observed in human patients, this could potentially increase the therapeutic window available for treatment decreasing CM-associated mortality and neurological sequelae. In combination with anti-parasite chemotherapy, molecules that stabilize plasma membranes and reduce overproduction of deleterious MP and shedding may be protective or minimise the cerebral complications associated with CM.

## Materials and Methods

Infections were performed as previously described [34] [37] [38]. All protocols adhered to the Australian Code of Practice for the Care and Use of Animals for Scientific Purposes. All protocols were approved by the Animal Ethics Committee of the University of Sydney (K20/7- 2006/3/4434 and K00/10-2010/3/5317).

### Detection and characterisation of plasma MP in mice with CM and NCM

#### i. Mice and infection

Seven to 8 weeks old female CBA mice (Animal Resource Centre, Perth) were housed under pathogen-free conditions. These mice are susceptible to *Plasmodium berghei-*ANKA (PbA) infection, and thus succumb to CM during the neurological phase, between day 6 and 14 post-infection (p.i) [37]. We induced CM in mice by intraperitoneal injection of 1×10^6^ PbA-parasitised red blood cells, as previously described [32] and 1×10^6^ *Plasmodium berghei*-K173 (PbK). NCM in mice was induced by infection with 2×10^6^ PbK-parasitised red blood cells [34]. It is noteworthy that the PbA genome and pathogenicity are close to *Plasmodium falciparum*, the causative agent of hCM, whereas PbK results in different pathogenicity (resembling *P. vivax*). Moreover, the early cytokine profile is different with increased IFN-γ production related to inoculum conferring protection against CM being observed only during PbK infection [77].

Parasitaemia was determined from thin tail blood smears on day 4 p.i and every second day until end point, using light microscopy and Diff-Quick staining. CM was diagnosed if an infected mouse presented with ruffled fur, severe motor impairment (ataxia, hemiplegia or paraplegia) or convulsions and was allocated a score of 3 or 4 as described previously [38]. Each mouse was also evaluated for the severity of CM using the clinical evaluation score [38]. Diagnosis was then confirmed by histopathology. Infected mice without any of the above mentioned symptoms were classed as NCM.

#### ii. Blood sampling and processing for MP analysis

Mouse venous blood was collected by retro-orbital venepuncture under anaesthesia in 0.129 mol/L sodium citrate (ratio of blood to anticoagulant 4∶1). Samples were centrifuged at 1 500 g for 15 min at room temperature. Harvested supernatant was further centrifuged at 18 000 g for 4 min, twice, to achieve platelet-free plasma (PFP).

#### iii. MP characterisation

Total MP numbers were quantified by detection of PS using FITC-Annexin V (Beckman Coulter) labelling, which is Ca^2+^ dependent. The cellular origin of these MP was determined using cell-specific monoclonal antibodies as detailed in Table 2.

**Table 2:** Detection of cell-specific markers.

Cell-type	Marker	Alternate name	Clone	Supplier	Concentration used
Endothelial cell	CD105	Endoglin	MJ7/18	eBioscience	5 µg/mL
Erythrocyte	TER-119/CD235a	Erythroid cell marker	TER-119	eBioscience	2 µg/mL
Leucocyte	CD45	Leucocyte Common Antigen	30-F11	Becton Dickinson Pharmingen	2 µg/mL
Monocyte	CD11b	Integrin αM	M1/70	eBioscience	2 µg/mL
Platelet	CD41	Integrin α IIb chain	MWReg30	Becton Dickinson Pharmingen	5 µg/mL
Intercellular Adhesion Molecule-1	CD54	ICAM-1	YN1/1.7.4	eBioscience	10 µg/mL
Vascular Cell Adhesion Molecule-1	CD106	VCAM-1	429	eBioscience	10 µg/mL

Briefly, 20 µl of PFP were incubated with Annexin V-FITC diluted 1 : 2 in 10× binding buffer (BB) or antibodies for 30 min. Following incubation, 20 µL of Flow-count™ Fluorospheres (1000/µL) (Beckman Coulter) were added to each sample to act as a calibrated internal standard of known size and concentration. Samples were resuspended in 200 µL of 1× BB and analysed on a Beckman-Coulter FC500-MPL flow cytometer. Data were acquired for 60 s and analysed using CXP analysis software (Beckman Coulter). MP were first discriminated based on their size (<1 µm) on a log-forward light scatter and log-side light scatter (FSC-SSC) dot plot and then for their positivity for binding of either Annexin V or specific antibodies (Fig. 2A). Due to their large size (10 µm) and high fluorescence, flow count beads could be discriminated from the MP population and gated accordingly.

### Clearance and tissue distribution of MP following adoptive transfer

#### i. MP purification and preparation

Blood was collected by venepuncture of the retro-orbital plexus in sterile citrated tubes from healthy and PbA infected mice displaying full blown syndrome. In order to maximise the number of MP purified without changing the phenotype of MP produced by the blood cells and to minimise the number of donor mice, whole blood was activated using calcium (Ca^2+^)-ionophore (Calcimycin A23187 2 mmol/L, SIGMA) vortexed and incubated at 37°C for 40 min. Calcium ionophore activation of whole blood increases the number of MP by two – three fold. Blood was processed for PFP as mentioned above. To obtain a purified population of MP devoid of blood proteins and calcium ionophore, PFP was further centrifuged at 18 000 g for 1 h at 15°C. Supernatant was used as a MP-free control after checking by flow cytometry. The MP pellet was gently resuspended in Diluent C (Sigma), prior to labelling using PKH67 Green Fluorescent Cell Linker Kit for General Cell Membrane Labelling (SIGMA). Briefly, under dark sterile conditions 1 µL of PKH67 was added to 250 µL of Diluent C and then added to 250 µL of MP suspension. Following continuous pipetting for 1 min, the suspension was incubated in the dark for 4 min. Labelling was stopped by adding 2 mL of foetal bovine serum (FBS) and 10 mL of RMPI-1640 containing 10% FBS (Gibco). The suspension was centrifuged at 18 000 g for 1 h at 15°C to pellet MP. PKH67^+^MP were resuspended in PBS and numbers were calculated after flow cytometry analysis.

#### ii. Adoptive transfer and detection of transferred MP in circulation by flow cytometry

PKH67^+^MP suspensions and MP-free supernatant were injected intravenously into healthy and PbA infected recipient mice 6 days p.i. Briefly, mice were anesthetised and received 400×10^3^ MP in 200 µL of PBS. Mice were allowed to recover. Blood was collected via tail vein at selected time points (1, 2, 3, 4, 5, 10, 20, 30, 40, 50 and 60 min) and analysed by flow cytometry prior to euthanasia and subsequent collection of organs. Briefly, 5 µL of mixed blood and citrate and 5 µL of Flow-count fluorospheres (Beckman Coulter) were resuspended in 200 µL of PBS. MP present in the samples were counted based on their size and PKH67 labelling.

#### iii. Tissue collection and immunofluorescence imaging

Following euthanasia, brains were collected and cut along the sagittal plane. By placing a small (1 mm×1 mm) section of fresh brain between two glass microscope slides and pressing these together, brain smears were created. Smears were allowed to air dry completely prior to fixation and subsequent labelling. The rest of the brain, together with lung, spleen, liver, kidney and heart tissue, were placed in cryoprotective embedding medium (OCT) and snap frozen in hexane cooled with liquid nitrogen. Tissue was then cut into 5 µm sections. Sections and brain smears were fixed in precooled (30 min at −20°C) absolute ethanol and acetone (3 : 1) for 10 min. Following blocking with filtered 5% (w/v) bovine serum albumin in PBS for 20 min, slides were incubated with Texas Red labeled Lycopersicon Esculentum (Tomato) Lectin (LEL, TL, Vector Laboratories) for 45 min at room temperature. Slides were washed and stained with 4′, 6-diamidino-2-phenylindole (DAPI) fluorescent stain (Invitrogen) and mounted in Fluoromount-G (Southern Biotech). Images were obtained using an Olympus IX71 inverted microscope and also the confocal microscope Olympus FV1000, as noted.

### Passive transfer of *in vitro* generated EMP

#### i. Cell culture, EMP generation, characterisation and transfer

EMP were generated from mouse brain microvascular endothelial cells (B3 cell line) *in vitro*, isolated as described previously [39]. Cells were maintained in RPMI 1640 (Gibco) supplemented with 10% FBS at 37°C in a 5% humidified CO_2_ incubator. Cells were grown to subconfluence and were stimulated with TNF (50 ng/mL) overnight (TNF-stimulated EMP, TNF-EMP). The supernatant was collected from multiple flasks, pooled and centrifuged to pellet MP, which were resuspended in PBS. Briefly, cells in suspension and large debris were eliminated by 1 800 g centrifugation for 10 min, then the supernatant was twice further centrifuged at 18 000 g to eliminate traces of the original culture medium and the final pellet was resuspended in sterile PBS. For controls, we used MP purified from supernatant collected from resting cells (non-stimulated MP (NS-EMP)) and Fluoresbrite Yellow Green Microspheres 0.75 µm (Polysciences, Inc.) (beads). Samples of EMP were phenotyped by incubation with anti-mouse monoclonal antibodies directed against CD54 (ICAM-1), CD105 (endoglin), CD106 (VCAM-1) and analysed by flow cytometry. Before transfer, EMP were labelled with PKH67, as described earlier, and counted based on PKH67 positive events falling under 1 µm in size.

Recipient mice were separated into two groups, healthy and PbA infected. PbA infected mice were infected 5 days prior to the EMP purifications to allow MP and mice to be ready at the same time. These groups were further divided to account for the experimental conditions (*n* = 3 per group). On day 4 p.i, mice were intravenously injected with PBS, inert microspheres (400×10^3^ beads/mouse), NS-EMP (400×10^3^ MP/mouse) or TNF-EMP (400×10^3^ MP/mouse). All mice were monitored, and the kinetics of clearance was measured for 60 min using flow cytometry, as described earlier. All mice were sacrificed on day 7 and brain and lung tissue were harvested for histological analysis.

#### ii. Histology

Formalin fixed, paraffin embedded brain and lung were Haematoxylin-Eosin stained. Slides were imaged at 100× magnification using an Olympus IX71 inverted microscope. Qualitative assessment of the tissue was performed by two independent researchers using parameters stipulated in the histopathological scale (Table 3). The brain was assessed for signs of oedema, haemorrhages and sequestered cells in vessels, and the lung was scored for cellularity in the alveolar septa, atelectasis, and intra-alveolar plasma and haemorrhages.

**Table 3:** The qualitative histopathological scoring of the tissue.

		Score				
Tissue	Parameter	0	1	2	3	4
**Brain**	Sequestration	None present	*	**	***	****
	PVS	No changes	*	**	***	****
	Haemorrhage	None present	*	**	***	****
**Lung**	Cellularity	None present	*	**	***	****
	Atelectasis	None present	*	**	***	****
	Plasma	None present	*	**	***	****
	Haemorrhage	None present	*	**	***	****

### Statistical analysis

Data were analysed using GraphPad Prism version 5.00 for Windows, GraphPad Software, San Diego California USA. Survival curves were analysed using the Log-rank (Mantel-Cox) Test and the Gehan-Breslow-Wilcoxon Test. To compare several groups, we used non-parametric analysis of variance (ANOVA, Kruskall-Wallis) with a Dunn's post-test. To compare mean total and cell-specific MP levels between two groups the Wilcoxon test was used; *p<0.05, **p<0.001, ***p<0.0001.

## Supporting Information

Figure S1 **High levels of PKH67-labelled MP (green) localised in the spleen following adoptive transfer, with less to none distributed in the brain, lung, liver, kidney and heart.** Cryosections were prepared from recipient mice with CD105-PE-labelled vessels (red) and DAPI-labelled nuclei (blue). Arrows indicate trapped MP, magnification (×400). Spleen (S), lung (L), kidney (K), liver (Li), brain (B) and heart (H).
(PDF)
