# Correction: Production, Fate and Pathogenicity of Plasma Microparticles in Murine Cerebral Malaria

**DOI:** 10.1371/journal.ppat.1004119

**Published:** 2014-04-14

**Authors:** The PLOS Pathogens Staff

The fourth and fifth authors, Georges E. R. Grau and Valery Combes, should be noted as having contributed equally to this work.
