# Supplementary figures and images for: Production, Fate and Pathogenicity of Plasma Microparticles in Murine Cerebral Malaria
Source: PLoS Pathog. 2014 Mar 20;10(3):e1003839. doi: 10.1371/journal.ppat.1003839 (PMC3961352; doi:10.1371/journal.ppat.1003839)

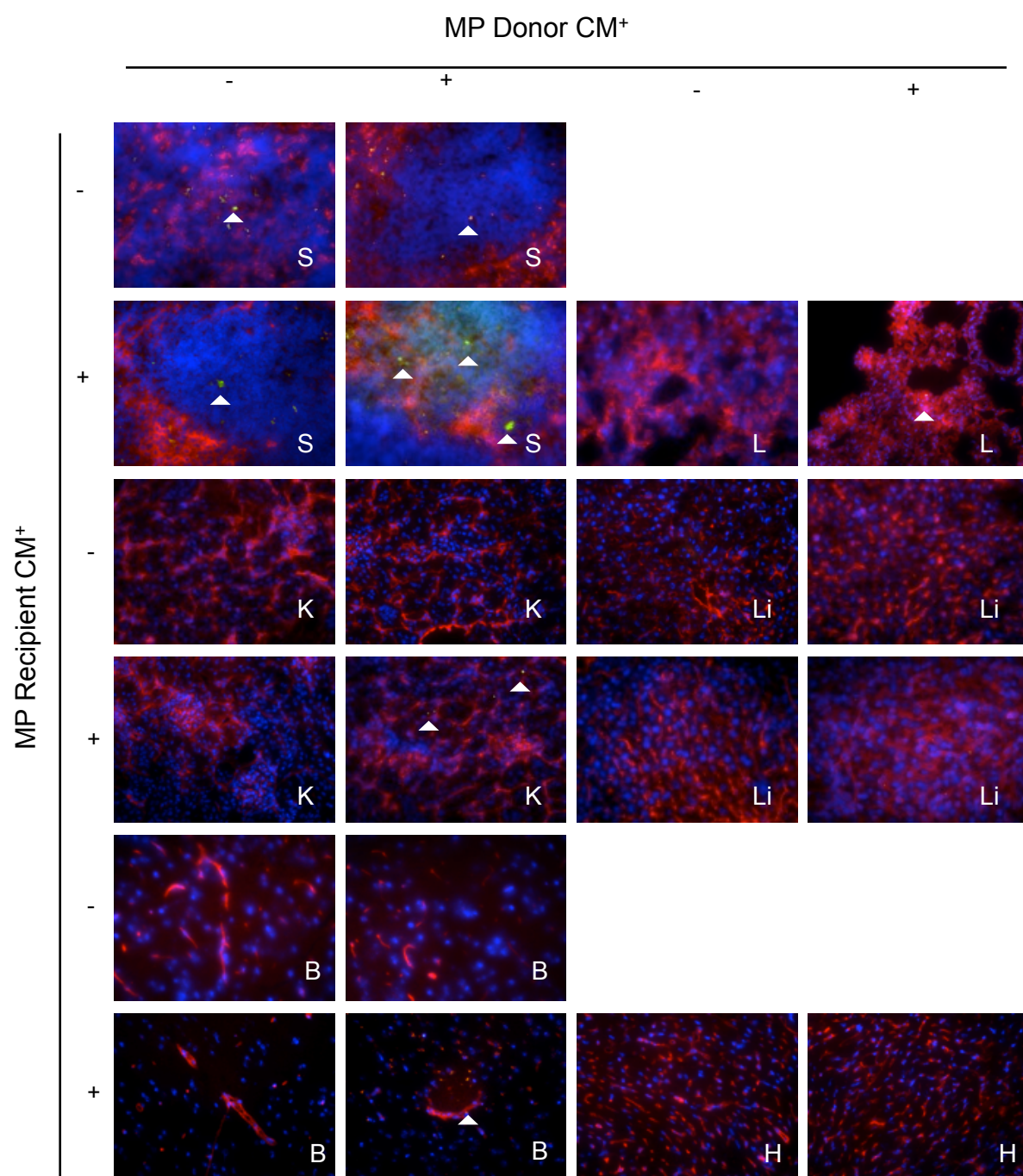

Supplement: Figure S1 — High levels of PKH67-labelled MP (green) localised in the spleen following adoptive transfer, with less to none distributed in the brain, lung, liver, kidney and heart. Cryosections were prepared from recipient mice with CD105-PE-labelled vessels (red) and DAPI-labelled nuclei (blue). Arrows indicate trapped MP, magnification (×400). Spleen (S), lung (L), kidney (K), liver (Li), brain (B) and heart (H). (PDF) [file ppat.1003839.s001.pdf]
